# Effect of Antigen Structure in Subunit Vaccine Nanoparticles on Humoral Immune Responses

**DOI:** 10.1021/acsbiomaterials.2c01516

**Published:** 2023-02-27

**Authors:** Jaeyoung Park, Julie A. Champion

**Affiliations:** School of Chemical and Biomolecular Engineering, Georgia Institute of Technology, 950 Atlantic Dr. NW, Atlanta, Georgia 30332-2000, United States

**Keywords:** subunit vaccine, nanoparticles, antigen structure, humoral immune response, salt precipitation

## Abstract

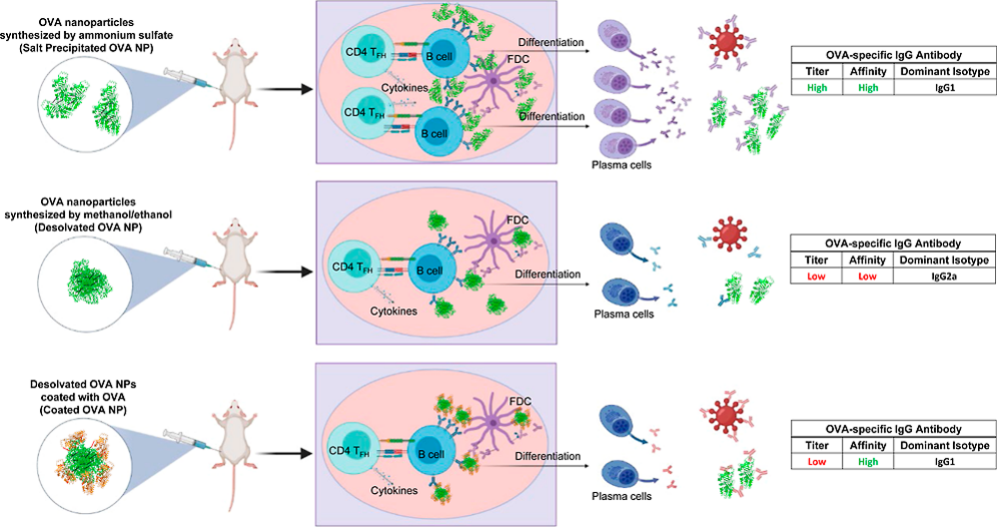

Subunit vaccines offer numerous attractive features, including good safety profiles and well-defined components with highly characterized properties because they do not contain whole pathogens. However, vaccine platforms based on one or few selected antigens are often poorly immunogenic. Several advances have been made in improving the effectiveness of subunit vaccines, including nanoparticle formulation and/or co-administration with adjuvants. Desolvation of antigens into nanoparticles is one approach that has been successful in eliciting protective immune responses. Despite this advance, damage to the antigen structure by desolvation can compromise the recognition of conformational antigens by B cells and the subsequent humoral response. Here, we used ovalbumin as a model antigen to demonstrate enhanced efficacy of subunit vaccines by preserving antigen structures in nanoparticles. An altered antigen structure due to desolvation was first validated by GROMACS and circular dichroism. Desolvant-free nanoparticles with a stable ovalbumin structure were successfully synthesized by directly cross-linking ovalbumin or using ammonium sulfate to form nanoclusters. Alternatively, desolvated OVA nanoparticles were coated with a layer of OVA after desolvation. Vaccination with salt-precipitated nanoparticles increased OVA-specific IgG titers 4.2- and 22-fold compared to the desolvated and coated nanoparticles, respectively. In addition, enhanced affinity maturation by both salt precipitated and coated nanoparticles was displayed in contrast to desolvated nanoparticles. These results demonstrate both that salt-precipitated antigen nanoparticles are a potential new vaccine platform with significantly improved humoral immunity and a functional value of preserving antigen structures in vaccine nanoparticle design.

## 1 Introduction

Vaccination has been one of the most effective strategies to curb the spread of infectious diseases by inducing long-term immunity with high potency against viruses.^1,2^ Vaccine platforms can be classified into several types: whole pathogen vaccines, nucleic acid vaccines, and subunit vaccines. Whole pathogen vaccines are the most common and have shown high potency against viruses, but they require sensitive analytical tools and strict controls during production to prevent the risk of infection.^3−5^ In addition, due to the use of entire pathogens, they can induce off-target immune responses toward undesired or highly variable epitopes. This could promote antibody-dependent enhancement of viral infections,^6^ or render vaccines less or ineffective against viral mutations, necessitating the update of vaccines.^7−11^ In contrast, nucleic acid vaccines and subunit vaccines do not carry the risk of infection and can be designed to minimize off-target responses by using only specific epitopes. Nucleic acid vaccines, especially mRNA vaccines, have made great advancements in vaccine effectiveness against Covid-19 cases and severity. However, their limitations are the requirement of storage at −20 °C or lower temperatures for long-term stability and lack of control over protein antigen expression amount, duration, and location.^12−17^

Owing to the use of only proteins, subunit vaccines can overcome limitations associated with whole pathogen and nucleic acid vaccines. Despite these advantages, subunit vaccines tend to have lower vaccine immunogenicity than mRNA and whole pathogen vaccines, and thus the addition of adjuvant and nanoparticle (NP) delivery systems with tunable physicochemical properties have been developed to improve their efficacy.^4,18−24^ However, due to safety concerns associated with adjuvants, it is often desirable to improve the efficacy of subunit vaccines using NPs without additional components.^25−27^ There have been multiple approaches to fabricate NPs including polymeric NPs with antigens and virus-like particles (VLPs). Polymeric NPs can be engineered to control the release of antigens effectively and enhance cellular uptake.^28,29^ But the challenges with polymeric NPs include off-target immune responses to polymers itself,^30−33^ limited capacity to incorporate sufficient antigens, and unstable conformation of antigens that are prone to damage by organic solvents.^29^ Self-assembled protein NPs like VLPs are common vaccine candidates because they mimic the structure of native viruses but lack viral components to be infectious.^34−36^ With *de novo* designs, their immunoreactivity can be enhanced, rendering them effective vaccine nanoparticles. However, a major problem of VLPs is their off-target responses induced by additional proteins or peptides to form the self-assembled capsid structure. The unintended immune response against protein components of carriers can be dominant, attenuating immune responses against target antigens on the surface of VLPs.^37,38^

To minimize or eliminate off-target responses, an effort has been made to develop and improve subunit vaccines made by desolvation.^39,40^ Desolvation is a method to fabricate antigen-only NPs by adding desolvants such as alcohol or acetone to induce hydrophobic interactions between antigens to form nanoclusters. Antigen nanoclusters are stabilized by cross-linking to form NPs and a key advantage is that no additional peptides or materials are needed that could trigger off-target immune responses. The method enables precise control of NP size and monodispersity and exhibits good yields. However, maintaining the native structure of antigens is crucial for a robust humoral immune response and desolvation can compromise the structure of conformational antigens.^41−43^ To address this issue, folded antigens have been cross-linked to the surface of desolvated NPs to present a conformationally recognizable surface. For example, tetrameric-conserved M2e peptides, whose immunogenicity are not structurally dependent, were desolvated to form NP cores and the cores were coated with trimeric headless hemagglutinin (HA) stalks to preserve the native structure of HA.^39^ The coated NPs conferred robust cross-protection against different strains of influenza viruses. However, the efficacy of coated vaccine NPs can be largely affected by the coating efficiency. There is only a limited surface area available for antigens, and surface adsorption also can negatively affect protein structures. Therefore, such limitations spur the need for a new antigen-only vaccine platform that preserves antigen structures.

A chemical reaction, such as cross-linking, can be an effective approach to directly form subunit vaccine NPs without damaging the protein structure. Direct cross-linking of proteins to form protein NPs was previously shown by Dong *et al.*(44) In the study, genipin, a fluorescent cross-linker found in the *Genipa americana* fruit extract, was used to cross-link ovalbumin (OVA) and form OVA NPs. After direct cross-linking, no structural change in OVA was observed. Genipin-cross-linked OVA NPs were used to examine antigen delivery *in vivo*. However, no beneficial immune response was observed compared to soluble OVA unless the adjuvant chitosan was included in the NPs. Another chemical reaction-mediated synthesis of NPs was demonstrated using a tyrosine-rich reactive tag. Wilks *et al.* formed peptide vaccine nanoparticles for flu by inducing dityrosine reaction to cross-link matrix protein 2 ectodomain (M2e).^45,46^ The cross-linking reaction between tyrosine residues was catalyzed by six histidine residues in tag and nickel ions with an oxidizer, such as magnesium monoperoxyphthalic acid. Prophylactic immunity against a lethal influenza virus challenge was seen in mice immunized with tyrosine cross-linked M2e NPs. Furthermore, cross-linked M2e NPs elicited higher M2e-specific IgG and IgG2a titers than naïve control, while uncross-linked M2e peptides did not. This suggests that antigen NPs prepared *via* the chemical reaction can improve humoral immune responses.

In this study, desolvant-free methods were established using cross-linkers or concentrated ammonium sulfate solutions to fabricate immunogenic antigen NPs while maintaining the native structure of antigens. OVA, a commonly used immunogenic chicken protein allergen, was used as a model antigen to examine the efficacy of desolvant-free vaccine NPs compared to desolvated and coated NPs. Prior to NP fabrication, molecular dynamics (MD) simulation was performed *via* GROMACS to computationally evaluate the conformational change and thermodynamic stability of OVA in desolvent versus water. In concert with *in silico* evaluation of proteins, the *in vivo* comparison of antigen NPs sheds light on the significance of antigen structure in subunit vaccine NPs and also develops new particle fabrication methods to further augment the efficacy of carrier-free antigen NPs.

## 2 Materials and Methods

### 2.1 Materials

For NP synthesis, Endofit Ovalbumin (<1 EU/mg) was purchased from InvivoGen. To fabricate endotoxin-free NPs, endotoxin-free Dulbecco’s phosphate-buffered saline (PBS), and endotoxin-free ultrapure water were purchased from Millpore Sigma, and ammonium sulfate (ultrapure, ≥99%), 3,3′-dithiobis(sulfosuccinimidyl propionate) (DTSSP) powder, succinimidyl 2-((4,4′-azipentanamido)ethyl)-1,3′-dithiopropionate (SDAD), and Pierce bicinchoninic acid (BCA) protein assay kit were ordered from Thermo Fisher Scientific.

For *in vivo* experiments, horseradish peroxidase (HRP)-conjugated rabbit anti-OVA IgG and goat anti-mouse IgG, IgG1, and IgG2a were purchased from Southern Biotech, and Armenian hamster anti-mouse CD3ε (PerCP), rat anti-mouse CD8a (FITC), rat anti-mouse CD4 (APC/Cy7), rat anti-mouse IFN-γ (PE), rat anti-mouse IL-4 (PE/Cy7), Armenian hamster anti-mouse CD11c (APC/Cy7), Zombie Violet Viability Kit, brefeldin A (1000x), and monensin (1000x) were purchased from Biolegend. Trustain FcX (anti-mouse CD16/32) and rat anti-mouse CD86 (PE) antibodies were ordered from BioLegend. Rat anti-mouse MHC-II, permeabilization buffer (10x), ACK lysing buffer (1x), RPMI 1640 supplemented with HEPES and l-glutamine, 1-Step Ultra TMB-ELISA substrate solution, and ELISA stop solution were ordered from Thermo Fisher Scientific. Phorbol 12-myristate 13-acetate (PMA) (HPLC grade, ≥99%) and calcium ionophore (TLC grade, ≥98%) were purchased from Millipore Sigma. PepTivator Ovalbumin was purchased from Miltenyi Biotec. Formaldehyde solution was purchased from VWR.

### 2.2 Molecular Dynamics Simulation

To solvate OVA with alcohol solvent, included topology files (ITP) for ethanol (PubChem CID: 702) and methanol (PubChem CID: 887) were generated by LigPargen,^47−49^ which provided optimized potentials for liquid simulations all atom (OPLS-AA) force field parameters for the organic compounds. After the topology data were manually registered to the MD GROMACS directory, a solvation box of 50:50 (v %/v %) methanol–ethanol mixture was assigned with OPLS-AA force fields and refined by performing energy minimization. The empty space of the mixture was filled with water molecules using the TIP3 model. The simple steepest descent minimizer was used with the particle mesh ewald (PME) method for a maximum of 50,000 steps. Then, the alcohol mixture was equilibrated in constant number, volume, and temperature (*NVT*) and constant number, pressure, and temperature (*NPT*) ensembles at 298 K. A Parrinello Rahman barostat with 1 bar as the reference pressure and V-rescale thermostat were applied for isotropic pressure and temperature couplings, respectively. For the MD simulation, the leap-frog integrator was chosen with an integration step of 2 fs, and the LINCS algorithm was applied to constrain hydrogen bonds. After a simulation of 50:50 (v %/v %) methanol–ethanol mixture, OVA (PDB: 1OVA) was solvated with water or the alcohol mixture. The parameters from the OPLS-AA forcefield and TIP3 water model were assigned to prepare the topology file of ovalbumin in an alcohol box of 28 × 28 × 28 nm^3^. After the solvated system was assembled, energy minimization was first carried out to refine the structure of the protein, and the system was further stabilized by *NVT* and *NPT* equilibration using the aforementioned settings. Posre and LINCS were used to apply position restraints on the heavy atoms of ovalbumin and hydrogen bonds, respectively. In addition, proteins and non-proteins were defined as two coupling groups. Following the equilibration, the MD were simulated for 20 ns in the NPT ensemble using the leap-frog algorithm.^50^

### 2.3 Nanoparticle Fabrication

Desolvated OVA NPs were synthesized by adding 400 μL of 50:50 (v %/v %) methanol/ethanol mixture to 620 μg OVA proteins resuspended in 100 μL PBS dropwise at a rate of 1 mL/min with a syringe pump while being stirred at a speed of 600 rpm. After desolvation, OVA NPs were centrifuged at 14,000*g* for 10 min at 4 °C, and NP pellets were resuspended in 500 μL PBS and stabilized by DTSSP cross-linker at a final concentration of 50 ng/μL under constant stirring at 600 rpm for 1 h. The OVA NPs were then centrifuged at 14,000*g* for 10 min at 4 °C to remove the remaining DTSSP and reaction byproducts, and the NP pellets were resuspended in 500 μL PBS and sonicated with a probe for 1 s on/3 s off at 50% intensity 15 times.

The same method was used to fabricate salt-precipitated NPs except 10 mg/mL OVA protein was precipitated with a 1:4 volume ratio of protein solution to 4.1 M ammonium sulfate instead of alcohol. Salt-precipitated NPs were stabilized with DTSSP at a final concentration of 140 ng/μL and resuspended in 300 μL PBS before sonication. To obtain monodisperse salt-precipitated NPs, each 100 μL batch of salt-precipitated NPs was sonicated and centrifuged at 1250*g* for 5 min at room temperature to collect 85 μL of supernatant and remove the pellets containing large particles.

To form directly cross-linked NPs, 1 mg OVA protein suspended in 100 μL PBS was incubated with DTSSP at a final concentration of 140 ng/μL under constant stirring at 600 rpm for 35 min. Then, the cross-linking reaction was quenched by adding 1 M Tris-HCl at pH 8.0 to a final concentration of 50 mM. Three batches of directly cross-linked NPs were pooled and centrifuged at 20,000*g* for 40 min at 4 °C. The pellet was resuspended in 300 μL PBS and sonicated with a probe for 1 s on/3 s off at 50% intensity 15 times. After sonication, each 100 μL batch of the directly cross-linked NPs was centrifuged at 1250*g* for 5 min at room temperature to collect 85 μL of supernatant.

For the fabrication of coated OVA NPs, 500 μL desolvated NPs were incubated with 31 μL of 2 mg SDAD dissolved in 515 μL DMSO under constant stirring at 600 rpm for 30 min to react the NHS group of SDAD with lysine residues of OVA. The NHS groups were then quenched by 25 μL of 1 M Tris-HCl at pH 8.0 for 5 min. The NPs were centrifuged at 14,000*g* for 10 min at 4 °C to remove excess cross-linkers and Tris and resuspend in 500 μL of 1 mg/mL OVA in PBS. The diazirine group of SDAD was activated with long-wave UV light at 370 nm to stabilize adsorbed OVA proteins on desolvated NPs by forming covalent bonds with any amino acid side chains or backbone of OVA.

### 2.4 Nanoparticle Characterization

The size, polydispersity index (PDI), and ζ potential of OVA NPs in PBS were measured by dynamic light scattering (DLS) with a Malvern Zetasizer Nano ZS. For the measurement, a viscosity of 0.8882 cP and refractive index of 1.33 was used for PBS, while a refractive index of 1.45 was used to analyze OVA NPs at 25 °C. Three measurements were taken per each sample, run at a scattering angle of 173° with a laser beam wavelength of 633 nm. For the evaluation of NP yield, the BCA assay was used to measure the concentration of OVA NPs. To assess the secondary structure of OVA in NPs, circular dichroism (CD) was performed with a ChiraScan-plus CD spectrometer (Applied Photophysics) in a wavelength range of 200–260 nm. OVA NPs were diluted to 0.1 mg/mL with PBS, and 175 μL NP solution was loaded into a 0.5 mm one-piece stoppered quartz cuvette (Applied Photophysics). CD signals were measured and reported in millidegrees representing ellipticity of OVA proteins in NPs.

### 2.5 *In Vivo* Immunization and Sample Collection

Six BALB/C mice (3 male, 3 female, 6- to 8-weeks old) were intramuscularly administered in thigh muscles of the hind limb with 10 μg salt-precipitated NPs, desolvated NPs, or coated NPs, and boosted with an identical injection 4 weeks later. Before the administration of mice with OVA NPs, endotoxin levels were measured by using a ToxinSensor Chromogenic LAL Endotoxin Assay Kit (GenScript) to ensure that the levels remained below the endotoxin limit of 15 EU/mg.^51^

Blood samples were withdrawn from the jugular vein of 3–5% isoflurane anesthetized mice before injection and at 2-week intervals. Collected blood samples were allowed to clot at room temperature for 30 min and centrifuged in BD microtainer capillary blood collectors at 6000*g* for 5 min to separate the serum from the clot. On the 8th week, mice were euthanized by CO_2_ asphyxiation to harvest spleens and subiliac lymph nodes on both sides. All animal experiments were carried out in accordance with regulations and guidelines approved by the Georgia Institute of Technology Institutional Animal Care and Use Committee under approved protocol number A100467.

### 2.6 Determination of Serum Antigen-Specific Antibody Titers and Affinity

ELISA was performed to determine OVA-specific antibody titers in sera using the following method. Maxisorp 96 well immune assay plates (Nunc) were coated with 1 μg/mL OVA protein in PBS at 25 °C overnight. Each well was blocked with 200 μL of 1% BSA in PBS at 25 °C for 2 h. After washing the plates, each well was incubated with serially diluted sera at 25 °C for 1 h 100 μL of HRP-conjugated goat anti-mouse IgG, IgG1, or IgG2a with 1:5000 dilution was then added to each well to measure OVA-specific IgG, IgG1, and IgG2a titers. After incubation at 25 °C for 1 h, each well was washed 3 times followed by the addition of 100 μL of TMB to each well. The enzymatic activity of HRP was stopped by 100 μL of 0.5 M H_2_SO_4_ in each well and the optical density (OD) at 450 and 570 nm were measured. The endpoint titer of antibodies from each mouse was determined by calculating the reciprocal of the highest dilution that gives OD_450_-OD_570_ twice that of the pre-vaccination serum at the same dilution.

Antibody affinity was assessed by sandwich ELISA. Briefly, serum antibodies were diluted according to the determined endpoint titer to achieve the same concentration. Each well of immune assay plates was then coated with 100 μL of the diluted serum antibody, followed by incubation at 25 °C overnight. The plates were washed three times and blocked with 1% BSA at 25 °C for 2 h. After washing, each well was incubated with 100 μL of 1:10 serially diluted OVA protein for 1 h. Each well was washed again to remove any remaining OVA and incubated with HRP-conjugated anti-OVA IgG for 1 h. Finally, the plates were washed three times, incubated with TMB, and the reaction with HRP was stopped by 0.5 M H_2_SO_4_. OD_450_-OD_570_ was then measured to generate a saturation curve for each group of antibodies. The K_D_ values were determined by one site saturation binding analysis *via* Graphpad Prism 9.

### 2.7 Cell Surface and Intracellular Cytokine Staining

Harvested spleens or lymph nodes were gently triturated and strained through 70 μm strainers with a 1 mL syringe plunger to obtain single cells. The spleens or lymph nodes on the strainers were then rinsed with 5–10 mL of RPMI 1640 medium supplemented with HEPES, l-glutamine, and 10% FBS, and the single cells were centrifuged at 350*g*, 4 °C for 5 min. Lymph node cell pellets were resuspended in a 200 μL complete RPMI 1640 medium, while splenocytes were incubated in 1 mL 1x ACK lysing buffer for 9 min, followed by quenching the lysis with 9 mL complete RPMI 1640. Splenocytes were then centrifuged at 350*g*, 4 °C for 5 min, and resuspended in 5 mL complete RPMI 1640. For staining intracellular cytokines, 1 × 10^6^ single cells were seeded in each well of a round-bottomed 96 well plate, centrifuged at 350*g*, 4 °C for 5 min, and resuspended in 100 μL complete RPMI 1640 medium supplemented with 2 μL of reconstituted OVA Peptivator stock solution to stimulate OVA-specific T cells. For positive controls, 0.5 μL of 2 μg/mL PMA and 0.5 μL of 100 μg/mL calcium ionophore were added to each well containing 1 × 10^6^ single cells. After incubation at 37 °C for 3 h, 2 μL mixture of 50x brefeldin A, and 50x monensin was added to each well to retain cytokines within the endoplasmic reticulum during cell activation for another 3 h. Before staining cellular surface markers, cells were stained with a Zombie Violet Viability Kit for 30 min as per the manufacturer’s instructions. Cells were resuspended in 0.5 μL/10^6^ cells in Trustain FcX Plus blocking buffer to block Fc receptors on the cell surface. Cells were then centrifuged and washed with 1% BSA in 1x PBS. For staining T cells, cells were incubated with 2 μL/10^6^ cells PerCP anti-CD3, 1 μL/10^6^ cells FITC anti-CD8, and 1 μL/10^6^ cells APC/Cy7 anti-CD4 for 40 min. For staining DCs, cells without re-stimulation were incubated with 2.5 μL/10^6^ cells APC/Cy7 anti-CD11c, 2.5 μL/10^6^ cells PE anti-CD86, and 0.5 μL/10^6^ cells FITC anti-MHC II. Stained cells were washed again with 1% BSA and fixed with 3.7% formaldehyde for 30–40 min. To stain intracellular cytokines of T cells activated by OVA, cells were resuspended in 1x permeabilization buffer with 1.5 μL/10^6^ cells PE anti-IFN-γ and 5 μL/10^6^ cells PE/Cy7 anti-IL-4. Finally, all T cells and DCs were resuspended in 1% BSA and analyzed by Cytek Aurora flow cytometry (Cytek Biosciences). Populations of activated immune cells were analyzed with Flow Jo using gating strategies demonstrated in Figures S1 and S2.

### 2.8 Statistical Analysis

Statistical comparisons were performed using an unmatched ordinary one-way ANOVA comparison followed by Tukey’s post-hoc multiple comparison analysis. For comparisons between groups at different times, a two-way ANOVA comparison was performed. P values of less than 0.05 were considered statistically significant. Statistical significance was marked with asterisks as follows: (*) for *p* ≤ 0.05, (**) for *p* ≤ 0.01, (***) for *p* ≤ 0.001, and (****) for *p* ≤ 0.0001, (ns) for statistically non-significant differences. All data plotted with error bars represent mean values with a standard deviation. The analysis was performed using Graphpad Prism 9.

## 3 Results and Discussion

### 3.1 *In Silico* Simulation of OVA Conformation and Thermodynamics

**Figure 1:**
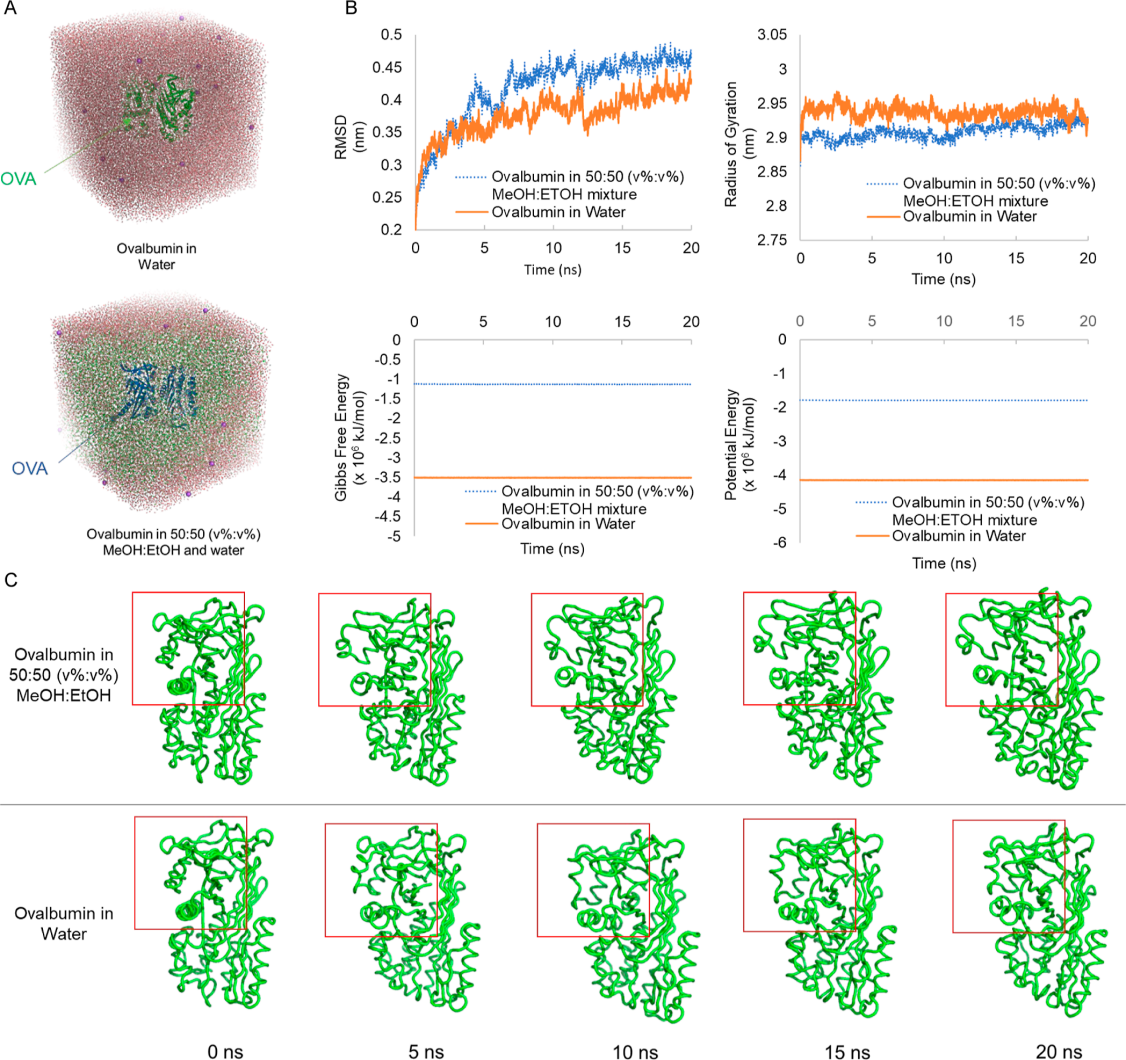
MD simulation of OVA proteins. (A) OVA proteins solvated with water (red) and 50:50 (v %/v %) methanol/ethanol (green). (B) Root mean square difference, radius of gyration, Gibbs free energy, and potential energy calculated from MD GROMACS. (C) Conformation of OVA simulated in the alcohol mixture and pure water for 20 ns. A part of OVA highlighted by a red box in the alcohol mixture shows a noticeable conformational change compared to that of OVA in water.

GROMACS was utilized to assess how the structure of OVA antigen changes in alcohol and water. GROMACS is an open-source software suite that can calculate and predict macroscopic properties of proteins at the atomic scale by using Newtonian equations of motion.^52^ Due to its versatile and accurate performance, it is one of the popular tools to predict properties of proteins, especially structural changes, and, therefore, is very useful for the assessment of OVA properties under different conditions.^52,53^ OVA was solvated with 50:50 (v %/v %) methanol–ethanol mixture, as used for the synthesis of desolvated NPs in this study, or pure water for comparison (Figure 1A). After the thermodynamics of solvated OVA were stabilized by NVT and NPT equilibration, each system was simulated for 20 ns using the OPLS-AA force field. Although the difference in compactness of OVA as measured by the radius of gyration was not significant, the root mean square difference (RMSD) of OVA, an indicator of change in structure coordinates, in 50:50 methanol–ethanol was slightly higher than that of OVA in water (Figure 1B). As shown in Figure 1C, OVA in a 50:50 alcohol mixture led to a greater shift in protein structure than OVA in pure water. In addition, significantly higher potential energy and Gibbs free energy were calculated for OVA in the 50:50 alcohol mixture than in water (Figure 1B), indicating less favorable thermodynamics associated with OVA in the alcohol mixture. Therefore, the results from the GROMACS simulation point to the instability of OVA protein structure in the alcohol mixture and demonstrates the negative impact of desolvation when forming antigen NPs.

### 3.2 Optimization and Characterization of OVA Nanoparticles

**Figure 2:**
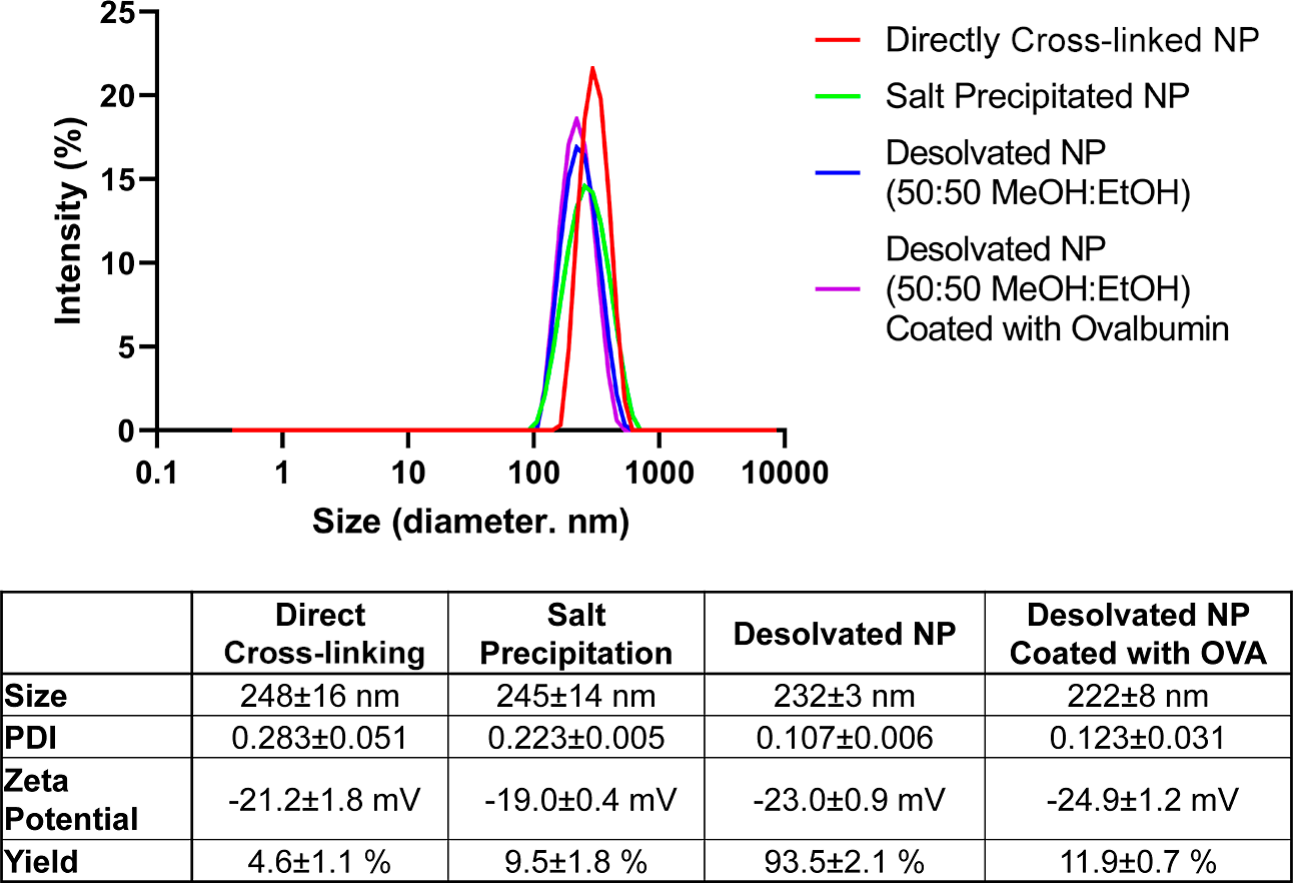
Physicochemical properties of NPs including particle size distributions and average diameter, polydispersity index (PDI), and ζ potential measured by DLS and Malvern ζ potential analyzer. The yield of each NP was calculated by measuring the amount of OVA proteins incorporated in each NP *via* BCA.

To examine the significance of antigen structure in NPs for eliciting potent humoral immune response, the protocols for the formation of desolvant-free NPs were established and optimized. Directly cross-linked OVA NPs were prepared by simply cross-linking soluble OVA under constant stirring at 600 rpm with amine reactive reducible cross-linker DTSSP. Salt-precipitated OVA NPs were fabricated by the addition of concentrated ammonium sulfate, a kosmotropic salt in the Hofmeister series, which induces clustering of protein *via* salting-out effects, while the protein structure remains intact,^54−56^ followed by stabilization by DTSSP cross-linker. To assess whether desolvated NPs with unfolded antigens could be “corrected”, they were coated with a layer of soluble OVA by a heterobifunctional SDAD cross-linker to generate coated OVA NPs in a manner similar to our previous influenza NPs.^39,57,58^ We also previously demonstrated that OVA-coated desolvated OVA NPs exhibited greater inflammatory responses than uncoated desolvated OVA NPs.^59^ All NPs were optimized to achieve similar physicochemical properties with a range of 220–250 nm in diameter and a ζ potential ranging from −19 to −25 mV (Figure 2). Desolvated and coated NPs were the most monodisperse, but desolvant-free particles had acceptable PDI values. Due to strong hydrophobic interactions as a driving force for desolvation, desolvated NPs were generated at the highest yield while the yield of directly cross-linked NPs was only ∼4.6%. With the help of ammonium sulfate, the yield was improved from 4.6 to 9.5%, and salt-precipitated NPs also exhibited a more monodisperse population than directly cross-linked NPs (Figure 2).

**Figure 3:**
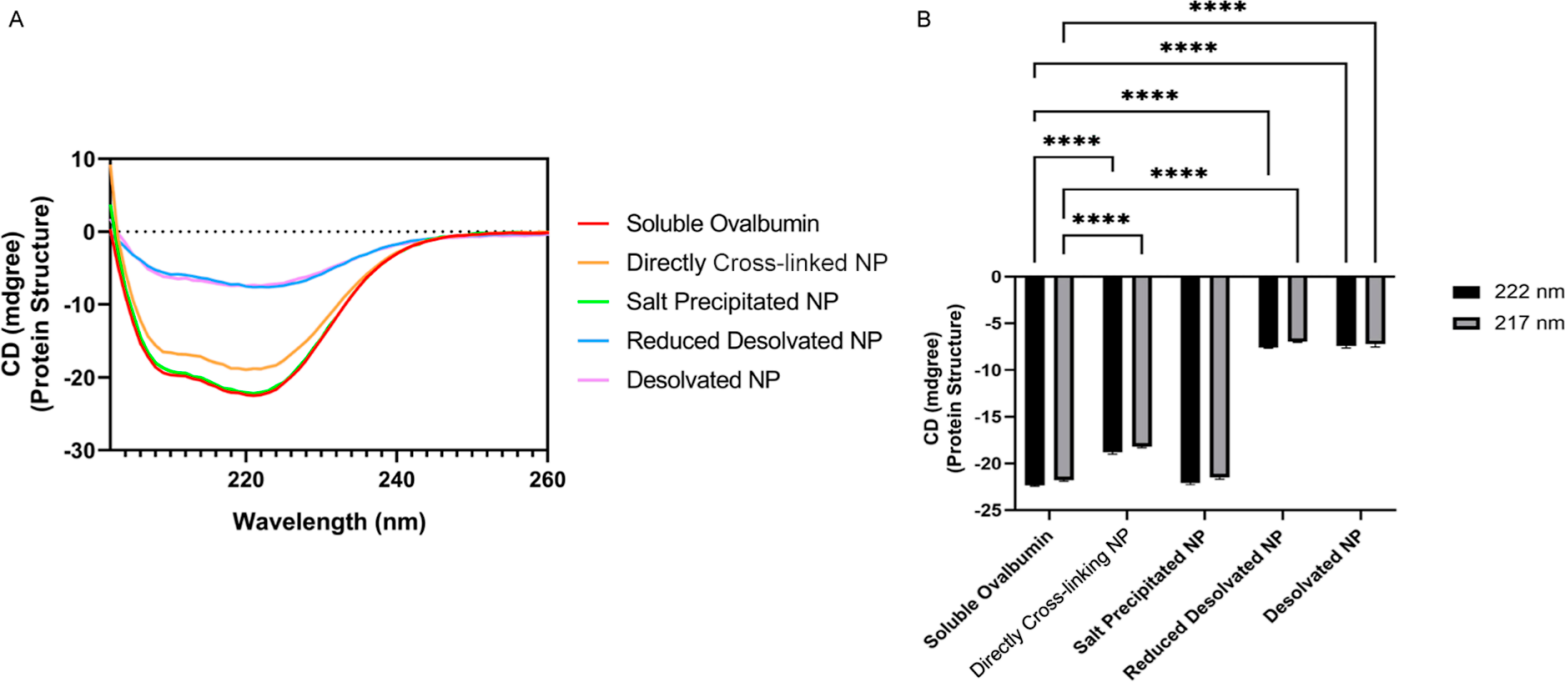
(A) Circular dichroism (CD) spectra of OVA NPs and soluble OVA and (B) CD signal intensity measured at 217 nm (β sheet) and 222 nm (α helix). *, *p* ≤ 0.05, **, *p* ≤ 0.01, ***, *p* ≤ 0.001, ****, *p* ≤ 0.0001.

While no remarkable discrepancy in physicochemical properties was observed, the difference in the OVA structure in NPs was distinguishable by CD. The ellipticity observed from salt-precipitated NPs was comparable to that from the native structure of soluble OVA, while a slightly reduced signal of ellipticity was observed from directly cross-linked NPs (Figure 3). This result, along with better yield and PDI of salt-precipitated NPs than directly cross-linked NPs, motivated the selection of salt-precipitated NPs for *in vivo* vaccination. As expected from the MD simulation, the signals for secondary structures, especially at 217 and 222 nm (Figure 3B), from desolvated NPs were significantly diminished, reflecting compromised structures of α helices and β sheets in desolvated OVA NPs. Reduction of the DTSSP bonds in desolvated NPs did not improve the signal. Signals from coated NPs were omitted because the amount of OVA proteins adsorbed on desolvated NP cores was not significant enough to detect the difference in OVA structures from coated and desolvated NPs. Hence, the results from CD were in alignment with MD simulations, demonstrating that the alcohol mixture can severely compromise protein structures. Furthermore, this study implies that concentrated ammonium sulfate can be utilized to form NPs while maintaining the native structure of proteins.

### 3.3 *In Vivo* Evaluation of Humoral Immune Response

**Figure 4:**
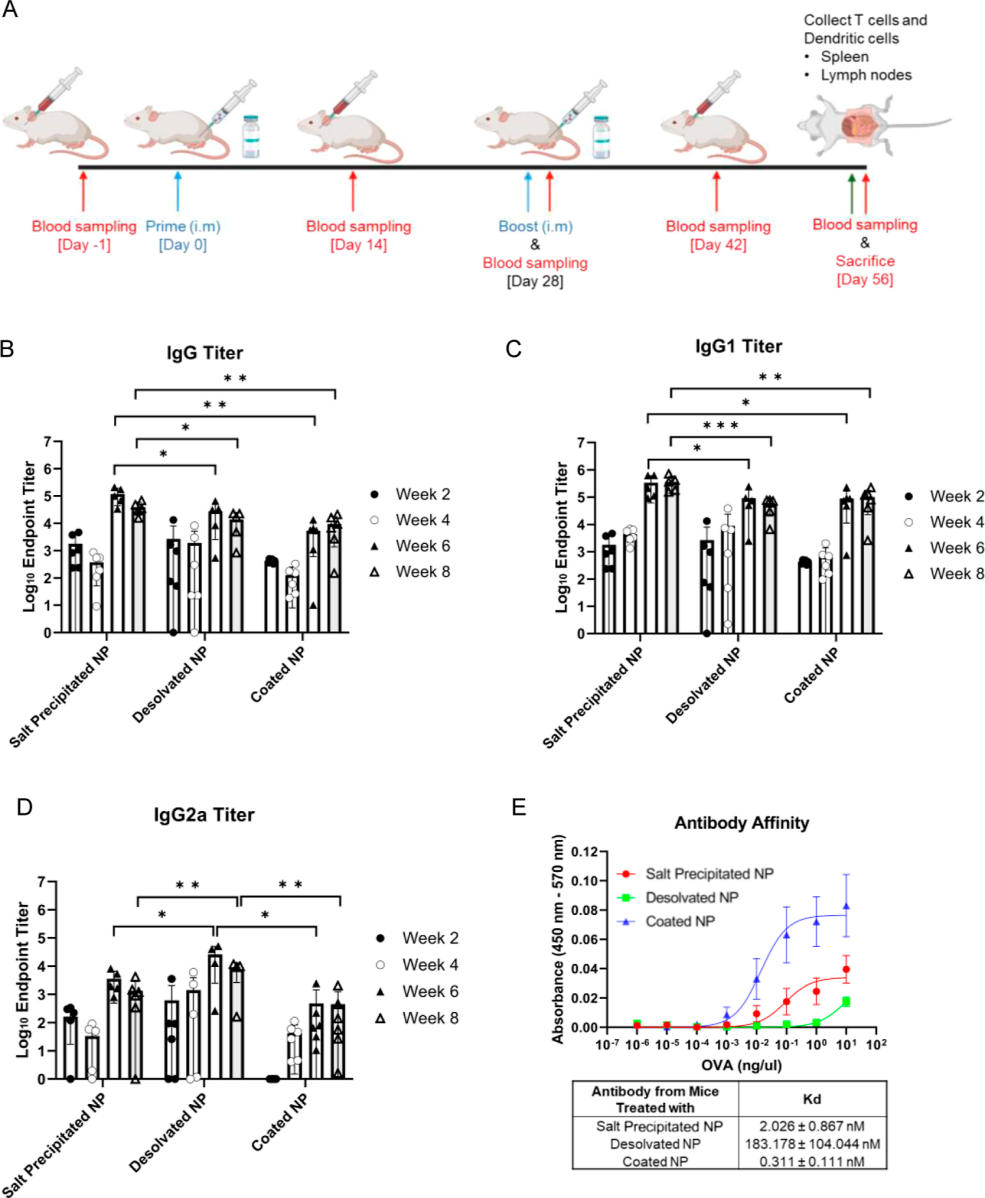
(A) Schematic overview of vaccination study. Total IgG (B), IgG1 (C), and IgG2a (D) from vaccinated mice after prime (circles) and after boost (triangles). (E) Antibody affinity of OVA-specific total IgG at 8 weeks. *N* = 5–6. *, *p* ≤ 0.05, **, *p* ≤ 0.01, ***, *p* ≤ 0.001, ****, and *p* ≤ 0.0001. ns represent statistically nonsignificant differences.

Owing to favorable physicochemical properties, salt-precipitated NPs were selected as a candidate for testing the significance of antigen structure on immune response, and their effectiveness was compared to desolvated and coated NPs. Soluble antigens were not included for animal studies as this study was designed to compare the effectiveness of different NPs presenting antigens in different structural context. We have previously shown that the model and influenza soluble antigens are less immunogenic than antigen nanoparticles.^57,59−62^ Each group of 6 mice was intramuscularly administered twice with 10 μg salt precipitated NPs, 10 μg desolvated NPs, or 10 μg coated NPs 4 weeks apart and serum was collected every 2 weeks. After each injection, there was no body weight loss (Figure S3). Primed mice did not show any sign of potent humoral immune response while the boost injection led to increased OVA-specific IgG titers (Figure 4). Salt-precipitated NPs induced a markedly stronger humoral immune response than desolvated NPs and coated NPs. As indicated in Figure 4B, vaccination with salt-precipitated NPs resulted in 4.2- and 22-fold greater OVA-specific IgG titers than desolvated and coated NPs in week 6, respectively. Although decreased sera IgG titers from all groups were observed in week 8 (Figure 4B), OVA-specific IgG titers collected in week 8 from mice vaccinated with salt-precipitated NPs was still the highest among different groups. A long term study is required to identify which types of nanoparticles can reactivate memory and provide long-term antibody responses.

Interestingly, salt-precipitated NPs strongly favored OVA-specific IgG1 titer (Figure 4C), which is associated with type 2 helper cell (Th2) or allergic response. Conversely, Th1-type response against intracellular pathogens was dominant for mice vaccinated with desolvated NPs, as evidenced by the highest OVA-specific IgG2a titers (Figure 4D). Coated desolvated NPs also skewed toward IgG1 production and Th2 response but, overall, antibody production was very low. It is worthwhile to note that ovalbumin is a well-known allergen for which the Th2 response or allergenicity can be largely altered by its conformational state.^63−65^ When OVA loses the tertiary structure, its allergenic response tends to be greatly reduced because antigenic determinants of OVA are only partially recognized by antibodies.^63^ Furthermore, it was reported that OVA conjugated onto the surface of NP micelles induced the production of high IgG1 titer with relatively low IgG2a titer.^66^ The same explanation may hold for the favored IgG1 response from mice vaccinated with salt-precipitated NPs, which best maintained the native structure of OVA. At the same time, denatured OVA in desolvated NPs might result in the inactivation of IgG1 binding epitope structures and make other hidden epitopes accessible, thus leading to the highest OVA-specific IgG2a titer though total OVA-specific IgG titer was low. It will be important to determine in future works if viral antigens presented in salt-precipitated NPs induce a Th1-biased response. Previously, we observed when flu subunit vaccines were formulated into NPs by desolvation, the use of specific antigenic sites and desolvation tended to affect Th1- and Th2-biased humoral immune responses. For example, NPs desolvated from three tandem copies of specific nucleoprotein antigenic peptides and coated with four tandem copies of matrix protein 2 ectodomain (4M2e) resulted in a higher 4M2e specific IgG2a/IgG1 ratio than NPs desolvated from native nucleoproteins coated with the same 4M2e.^57^ Additionally, desolvated HA NPs coated with additional HA antigens favored a Th2 response, suggesting desolvated HA antigens could have affected the isotype of antibodies.^58^ Therefore, salt-precipitated NPs will be useful to examine the effect of pathogen antigen structures on Th1- and Th2-associated immune responses.

In addition to titer, the affinity of OVA-specific IgG collected from mice at 8 weeks administered salt-precipitated NPs (*K*_D_ ≈ 2.026 nM) was significantly stronger than IgG from mice treated with desolvated NPs (*K*_D_ ≈ 183.178 nM) (Figure 4E). Interestingly, coated desolvated NPs induced the highest affinity OVA-specific IgG (*K*_D_ ≈ 0.311 nM). This implies that a layer of OVA proteins with a preserved structure was sufficient to engage interactions with B-cell receptors (BCRs) and achieve high affinity antibodies. These results further suggest that IgG titer may not simply be affected by the mass of antigens with preserved structures. As the same mass of NPs is injected into each mouse, salt-precipitated NPs were injected in greater numbers than desolvated and coated NPs due to higher density of desolvated NPs and coated NPs (Figure S4) relative to salt-precipitated NPs. This is likely because salt-precipitated proteins had less driving force to cluster compared to desolvated NPs. More salt-precipitated NPs, in turn, could provide a larger number of conformational epitopes available on the surface of NPs to directly interact with BCRs than densely coated desolvated NPs. Therefore, a larger number of activated B cells stimulated by salt-precipitated NPs could have undergone somatic hypermutation and differentiate into plasma cells to secrete antibodies. In contrast to improved IgG and IgG1 titers using salt-precipitated OVA NPs without adjuvants, Dong *et al.* demonstrated that genipin cross-linked OVA NPs, which also preserved the OVA structure, did not elicit significant OVA-specific IgG, IgG2a, or IgG1 titers compared to soluble OVA.^44^ However, the addition of chitosan as an adjuvant in the genipin cross-linked OVA NPs significantly enhanced titers of the three antibody isotypes. This implies that the method of NP synthesis may be critical for a humoral immune response even if different methods still maintain the native conformation of antigens.

### 3.4 *In Vivo* Evaluation of Cellular Immune Response

**Figure 5:**
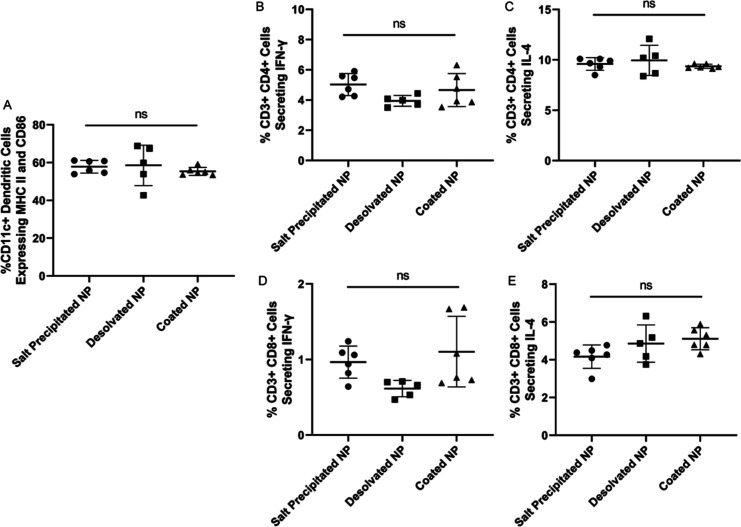
Percent population of (A) CD11c+ DCs expressing MHC II and CD86, OVA-specific CD4+ T cells secreting (B) IFN-γ and (C) IL-4, and OVA-specific CD8+ T cells secreting (D) IFN-γ and (E) IL-4 obtained from spleens. *N* = 5–6. ns represents statistically non-significant differences.

Though T cells are not activated by structural antigens, we analyzed populations of activated immune cells from spleens and inguinal lymph nodes to examine their effect on the humoral immune response. Approximately 50–60% of splenic DCs, marked by their expression of CD11c, expressed MHC II and CD86 (Figure 5A), indicating successful activation of DCs by OVA NPs. OVA-specific CD4+ and CD8+ T cells harvested from spleens were also analyzed by stimulating T cells with OVA Peptivator, a pool of OVA peptides consisting of 15-mer sequences. According to intracellular cytokine staining (Figure 5B,C) of activated CD4+ T cells, there was no statistically significant difference between percent populations of helper T cells secreting IFN-γ and IL-4, which are hallmarks of Th1- and Th2-type responses, respectively. Furthermore, there was also no evident difference in activated CD8+ T cell cytokine secretion (Figure 5D,E). This is consistent with our previous works where the administration of coated OVA NPs did not induce higher splenocyte cytokine production than saline-treated mice.^67^ Dong *et al.* also reported that genipin cross-linked OVA NPs without adjuvants did not enhance cellular immune responses compared to soluble OVA as evidenced by their similar percentages of restimulated CD8+ and CD4+ T cells from the spleens of OT-I mice.^44^ However, this effect may be antigen specific as desolvated M2e NPs did induce high splenocyte IFN-γ production,^62^ though M2e is a very small antigen relative to OVA with little secondary structure.^68^

**Figure 6:**
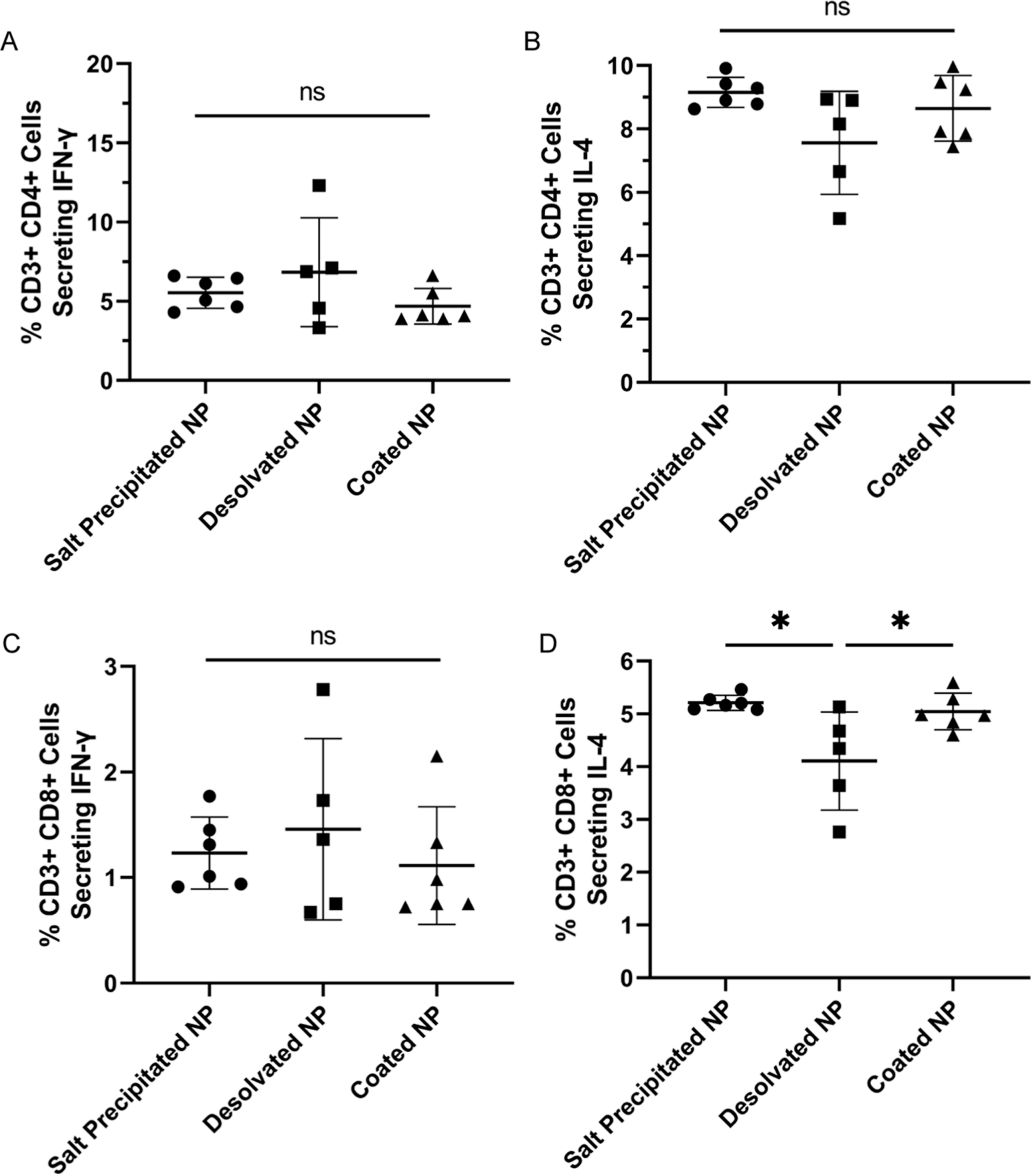
Percent population of OVA-specific CD4+ T cells secreting (A) IFN-γ and (B) IL-4 and OVA-specific CD8+ T cells secreting (C) IFN-γ and (D) IL-4 obtained from lymph nodes. *N* = 5–6. *, *p* ≤ 0.05. ns represents statistically non-significant differences.

Next, OVA-specific CD4+ and CD8+ T cells from lymph nodes were analyzed by conducting intracellular cytokine staining. As shown in Figure 6A,B, differences in the populations of OVA-specific CD4+ T cells secreting IFN-γ or IL-4 were not statistically significant. Interestingly, the population of OVA-specific CD8+ T cells secreting IL-4 (Figure 6D) activated by both salt precipitated or coated NPs was slightly higher than that from desolvated NPs. Given the role of CD4+ T cells in promoting the CD8+ T cell function and the IgG1 bias, one would expect an increase in the IL-4 secretion for CD4+ T cells in salt precipitation and coated NP groups compared to desolvated, though it was not observed. Still, all OVA NPs were able to induce similar levels of IL-4 cytokines from OVA-specific T cells and there was a larger fraction of IL-4 secreting CD4+ T cells than IFN-γ secreting CD4+ T cells in both spleen and lymph node populations. Collectively, these results indicate that the Th1-associated IgG2a response mounted by desolvated NPs was not primarily due to altered cellular responses but, rather, due to an altered antigen structure.

## 4 Conclusions

In this study, OVA NPs were fabricated *via* different methods, and their physicochemical properties were assessed after multiple rounds of optimization. *In silico* simulation and CD demonstrated that desolvation severely compromised the native structure of OVA while salt precipitated and directly cross-linked NPs successfully preserved the native structure. Upon vaccination of mice, both salt precipitated and coated OVA NPs improved the affinity of antibodies, suggesting that the preserved structure of antigens is critical for eliciting a humoral immune response with high affinity. Furthermore, salt-precipitated NPs mounted a much stronger humoral immune response than both desolvated and coated NPs. Although OVA-specific IgG titers from mice immunized with OVA NPs decreased in week 8, titers of total IgG and IgG1 elicited by salt-precipitated NPs were still the highest among the different groups. Interestingly, desolvated NPs shifted the dominant isotype of OVA-specific IgG from Th2 associated IgG1 to Th1 associated IgG2a, although there was no significant difference for a cellular immune response among different groups of NPs. This indicated that the antigen conformational change might affect IgG isotype titer by altering the antigenic sites to which certain antibody isotypes preferentially bind. Overall, this study demonstrates the use of salt-precipitated NPs as a potent vaccine candidate and stresses the significance of antigen structure in NPs to improve the effectiveness of subunit vaccine. However, the effect of antigen structures on the shift in targets of antibodies favored by certain isotypes will need to be further evaluated by carefully investigating antigens other than OVA.

## Supporting Information Available

Gating strategy for analyzing *in vivo* T-cell immune responses; gating strategy for analyzing *in vivo* dendritic cell population; body weights of immunized mice; and analysis of density of ovalbumin nanoparticles (PDF)
