# Supplementary material for: Effect of Antigen Structure in Subunit Vaccine Nanoparticles on Humoral Immune Responses
Source: ACS Biomater Sci Eng. 2023 Feb 27;9(3):1296–306. doi: 10.1021/acsbiomaterials.2c01516 (PMC10015428; doi:10.1021/acsbiomaterials.2c01516)
Supplement: Supplementary file 1 — ab2c01516_si_001.pdf [file ab2c01516_si_001.pdf]

Supporting Information

Effect of Antigen Structure in Subunit Vaccine Nanoparticles on Humoral Immune Responses

Jaeyoung Park[a, b], Julie A. Champion[a, c]

[a]*School of Chemical and Biomolecular Engineering, Georgia Institute of Technology, 950 Atlantic Dr. NW, Atlanta, GA, 30332-2000, USA.*

[b]first author: jpark842@gatech.edu

[c]corresponding author: julie.champion@chbe.gatech.edu

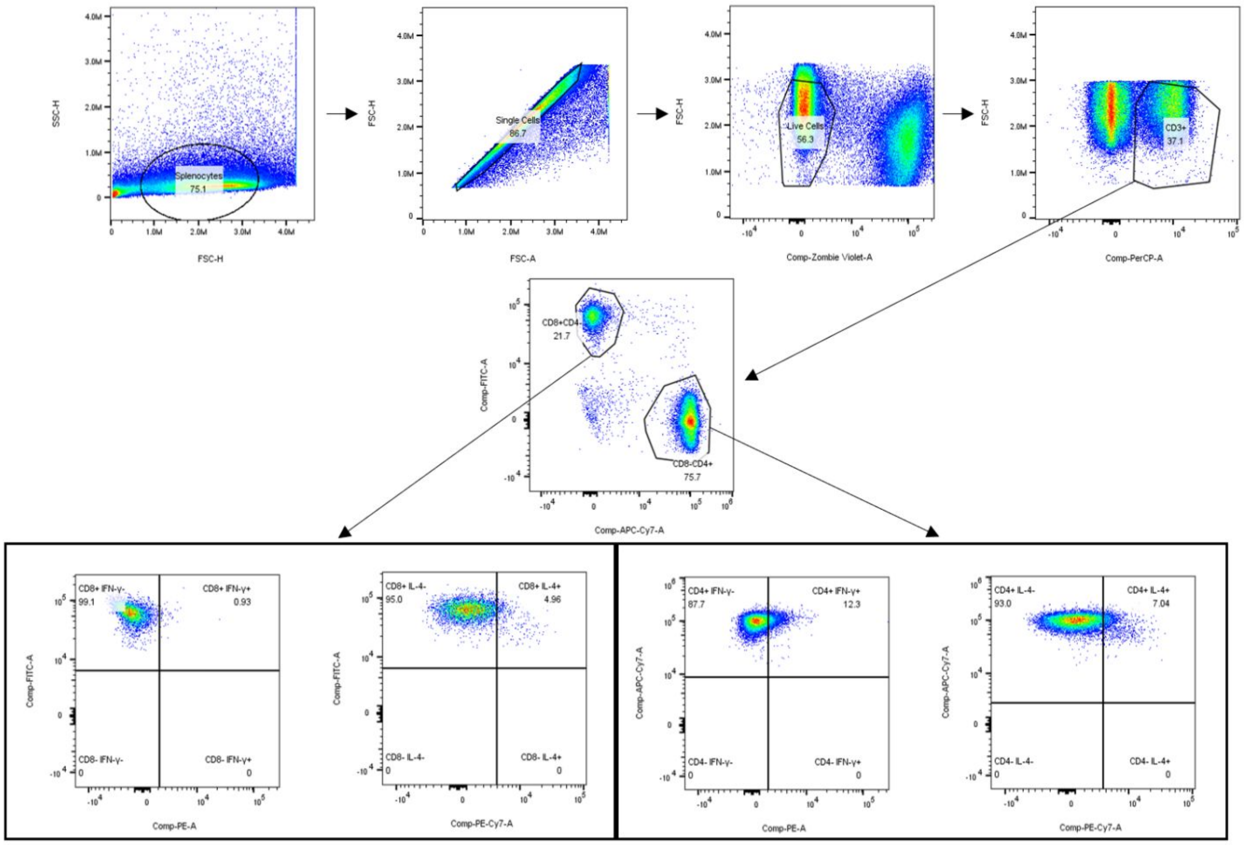

Figure S1. Gating of splenocytes for analyzing percent population of CD4+ and CD8+ T cells secreting IFN-γ and TNF-α.

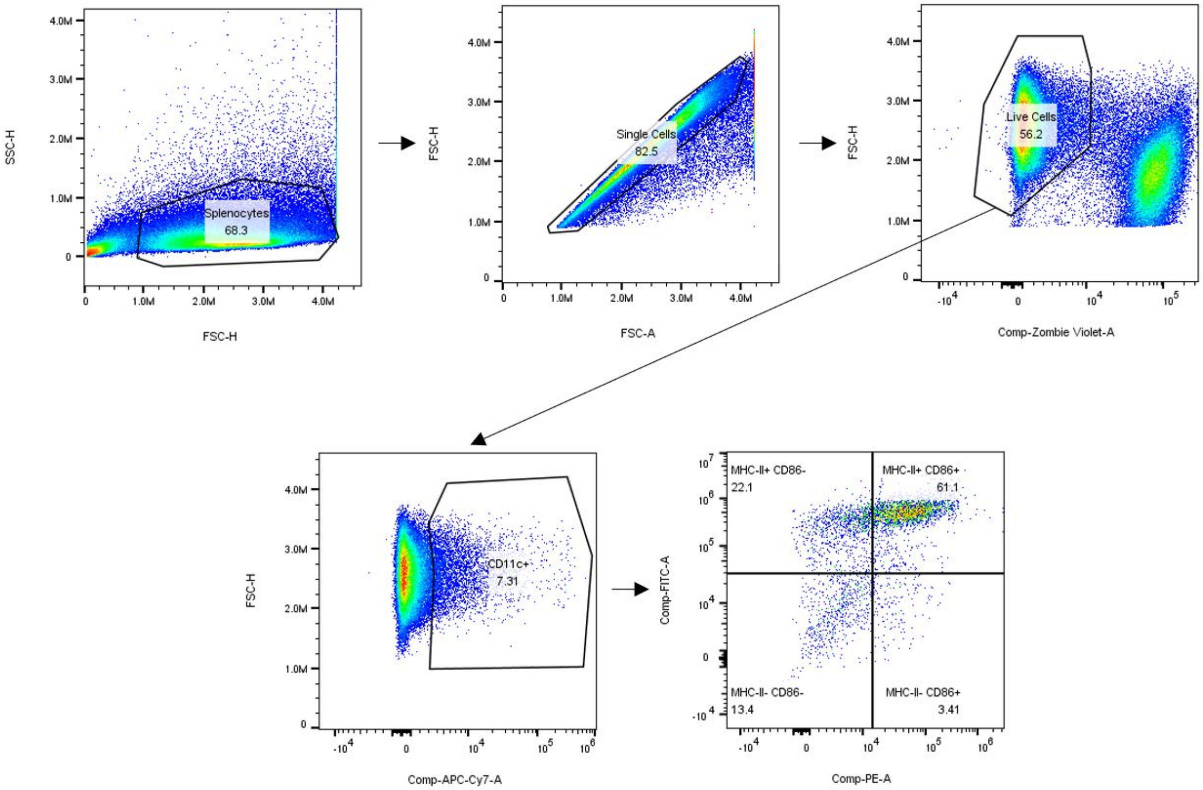

Figure S2. Gating of splenocytes for analyzing percent population of dendritic cells with expression of MHC-II and CD86 surface markers.

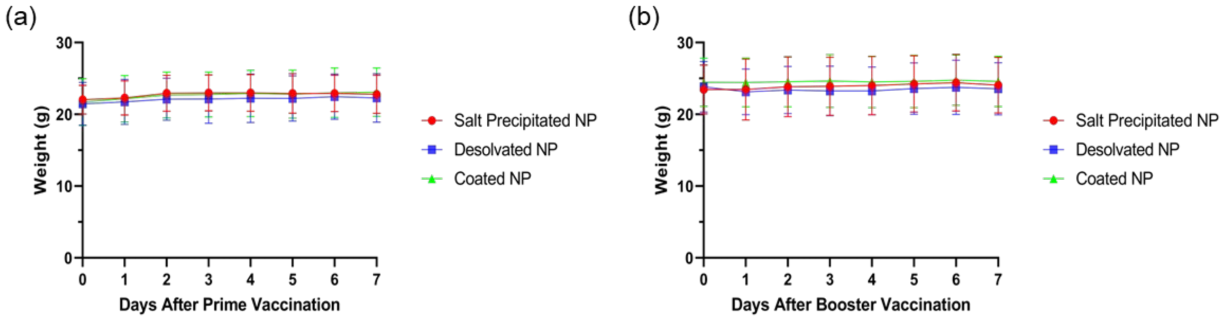

Figure S3. Body weights of mice administered OVA NPs monitored after (a) prime and (b) booster vaccination.

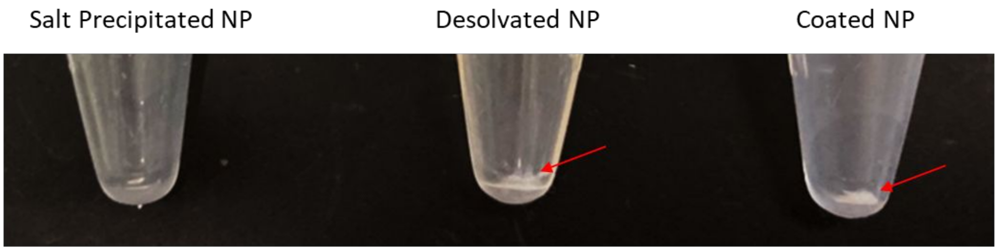

	Salt Precipitated NP	Desolvated NP	Coated NP
Mass before Centrifugation (7,000xg)	20 µg	20 µg	20 µg
Mass after Centrifugation (7,000xg)	0.08 µg	20 µg	16 µg

Figure S4. Comparison of density of OVA NPs. 20 µg of OVA NPs was centrifuged at 7,000xg, 25°C for 10 min. Compared to desolvated and coated NPs, pellets of salt precipitated NPs were barely detectable. Pelleted OVA NPs were resuspended in 45 µL 1x PBS to measure the mass of pelleted OVA NPs by BCA assay. The lowest amount of pelleted salt precipitated NPs indicate the lowest density of salt precipitated NPs, implying that the number of salt precipitated NPs was the highest among other OVA NPs at the same mass.
